# Different probiotic strains alter human cord blood monocyte responses

**DOI:** 10.1038/s41390-022-02400-5

**Published:** 2022-12-07

**Authors:** Xenia Rückle, Jessica Rühle, Leonie Judd, Janine Hebel, Stefanie Dietz, Christian F. Poets, Christian Gille, Natascha Köstlin-Gille

**Affiliations:** 1 Department of Neonatology, Tübingen University Children’s Hospital, Tübingen, Germany; 2 Department of Neonatology, Heidelberg University Children’s Hospital, Heidelberg, Germany

## Abstract

**Background:**

Probiotics have a protective effect on various diseases. In neonatology, they are predominantly used to prevent necrotising enterocolitis (NEC), a severe inflammatory disease of the neonatal intestine. The mechanisms by which probiotics act are diverse; little is known about their direct effect on neonatal immune cells.

**Methods:**

In this study, we investigated the effect of probiotics on the functions of neonatal monocytes in an in vitro model using three different strains (*Lactobacillus rhamnosus* (LR), *Lactobacillus acidophilus* (LA) and *Bifidobacterium bifidum* (BB)) and mononuclear cells isolated from cord blood.

**Results:**

We show that stimulation with LR induces proinflammatory effects in neonatal monocytes, such as increased expression of surface molecules involved in monocyte activation, increased production of pro-inflammatory and regulatory cytokines and increased production of reactive oxygen species (ROS). Similar effects were observed when monocytes were stimulated simultaneously with LPS. Stimulation with LA and BB alone or in combination also induced cytokine production in monocytes, with BB showing the least effects.

**Conclusions:**

Our results suggest that probiotics increase the defence functions of neonatal monocytes and thus possibly favourably influence the newborn’s ability to fight infections.

**Impact:**

- Probiotics induce a proinflammatory response in neonatal monocytes in vitro.
- This is a previously unknown mechanism of how probiotics modulate the immune response of newborns.
- Probiotic application to neonates may increase their ability to fight off infections.

## Introduction

Infections are one of the most common complications in preterm infant care. About one in three very low birth weight infants (VLBWI) suffers at least one infection during their stay in the neonatal intensive care unit (NICU).^1,2^ Most neonatal infections involve mucosal surfaces. For both, neonatal sepsis and necrotising enterocolitis (NEC), which are the most important infections in preterm infants, it has been shown that alterations in the intestinal microbiome precede disease onset and that causative pathogens often descend from the intestinal flora of the infected infant.^3–6^

Immediately after birth the newborn´s body surfaces are colonised with bacteria. Various factors such as mode of delivery, diet, antibiotic therapy and others influence microbiome establishment.^7–9^ Changes in the microbiome in the sense of dysbiosis seem to be associated with the occurrence of infections in preterm infants.^10–12^

More than 100 years ago, Elie Metchnikof already suspected that a modulation of the microbiome by probiotics could have a beneficial effect on human health.^13^ There is now evidence that probiotics have a protective effect on a wide range of diseases such as inflammatory bowel disease,^14^ allergy,^15^ obesity,^16^ autoimmune diseases^17^ and many others. In preterm infants, probiotics have been shown to reduce the incidence of NEC^18–20^ and late-onset sepsis (LOS).^19^

Various mechanisms have been described through which probiotics exert their protective effects, e.g. by promoting the formation of short chain fatty acids (SCFA) by bacteria of the microbiome,^21^ interfering with tryptophan metabolism,^22^ inducing the production of the immunoregulatory cytokine TGF-β in the intestine^23^ and promoting the generation of regulatory T cells (T_regs_)^24^ (reviewed in ref. ^25^)

In the study presented here, we aimed to investigate the effect of different probiotic strains used in neonatology on the in vitro immune response of neonatal monocytes. We chose three different probiotic strains for this purpose. The first strain, *Lactobacillus rhamnosus* (LR) was chosen because by far the most in vivo studies and clinical investigations exist for this probiotic strain.^26^ The other two strains, *Lactobacillus acidophilus* (LA) and *Bifidobacterium bifidum* (BB) were chosen because they are contained in a probiotic that is very commonly used in neonatology worldwide (Infloran®).^18,27–29^ LA and BB were investigated individually but also in combination, since there is evidence that combinations of *Lactobacilli* and *Bifidobacteriae* in particular have favourable in vivo effects in neonates.^30^ As target parameters, we chose surface markers associated with monocyte activation, expression of pro- and anti-inflammatory cytokines, and production of reactive oxygen species (ROS). We found that (1) stimulation with LR alone induced expression of molecules involved in monocyte activation on their cell surface, (2) increased production of pro-inflammatory and regulatory cytokines and (3) induced production of ROS by neonatal monocytes. (4) This proinflammatory effect of LR stimulation was also observed if used in combination with lipopolysaccharide (LPS). (5) Stimulation with LA and BB alone or in combination had similar effects on cytokine production of neonatal monocytes, with BB having the least stimulating activity.

## Methods

### Patients

Cord blood was collected from healthy term neonates (≥37 + 0 gestational weeks) immediately after primary Caesarean section. Children with intra-amniotic infection (defined by the German Society for Gynaecology and Obstetrics (DGGG) as maternal fever (≥38.0 °C), increased maternal inflammatory markers without any other cause (CRP > 10 mg/l or elevation of white blood cell count >15,000/μL), foetal or maternal tachycardia, painful uterus and foul-smelling amniotic fluid) were excluded. Parents gave written informed consent and the study was approved by the local ethics committee (248/2005A).

### Cell isolation and culture

Mononuclear cells (MNC) from heparinised cord blood (CBMC) were isolated by density gradient centrifugation according to a previously described protocol.^31^ Heparinised whole blood was diluted in phosphate-buffered saline (PBS) to a total volume of 35 ml and added carefully onto 15 ml lymphocyte separation solution (Biochrom GmbH, Berlin, Germany). Cells were centrifuged and the MNC layer was collected. Cell count was determined in a SYSMEX-KX21 cytometer (Sysmex GmbH, Norderstedt, Germany) and cells were diluted in RPMI 1640 (Pan Biotech, Aidenbach, Germany) supplemented with 10% foetal calf serum (FCS) and 1% glutamine. Cells were set to a final concentration of 2 × 10^6^ cells/ml and cultured in a 48-well plate overnight prior to stimulation with probiotics (0.5 ml/well).

### Preparation of probiotic strains

A clinical isolate of *Lactobacillus rhamnosus* and the probiotic strain *Lactobacillus acidophilus* (DSM20079) were kindly provided by Prof. Matthias Marschal (Institute for Microbiology and Hygiene, University Hospital Tübingen, Germany). The probiotic strain *Bifidobacterium bifidum* (DSM20456) was kindly provided by the Leibniz institute DSMZ, German collection of microorganisms and cell cultures GmbH. Strains were stored as stocks at −80 °C. For in vitro experiments, bacteria were plated on agar plates and incubated at 37 °C for 3–4 days. LR was cultured on Columbia Sheep Blood Agar plates (Mibius, Düsseldorf, Germany) under aerobic conditions, LA on De Man, Rogosa and Sharpe (MRS) Agar (Mibius, Düsseldorf, Germany) under anaerobic conditions (Thermo Scientific™ Oxoid™ AnaeroGen™ 2.5l-bag, Thermo Fisher Scientific Inc., Waltham) and BB on Bifidobacterium Agar (Aurosan GmbH, Essen, Germany) under anaerobic conditions (Thermo Scientific™ Oxoid™ AnaeroGen™ 2.5l-bag). After three to four days of culture, colonies were picked and washed two times in PBS by centrifugation at 16,000 × *g* for 3 min.

A dilution series was prepared before the start of experiments. For stimulation experiments LR, LA and BB were used at a multiplicity of infection (MOI) of 1: 0.1 and 1:1.

### Simulation of CBMC with probiotics

For analysis of expression of surface molecules and for analysis of ROS production after stimulation with LR, LR diluted in PBS was added in a MOI of 1:0.1 and 1:1 to 10^6^ CBMC in RPMI supplemented 10% FCS and 1% glutamine and cultured for 1 h.

For analysis of cytokine expression after probiotic stimulation, LR, LA, BB or a combination of LA and BB diluted in PBS was added in a MOI of 1:0.1 and 1:1 to 10^6^ CBMC in RPMI supplemented 10% FCS and 1% glutamine and cultured for one hour. Afterwards, 10 µg/ml Brefeldin A (Sigma, Steinheim, Germany) alone or in combination with different concentrations of lipopolysaccharide (LPS, Sigma, 1 µg/ml, 100 ng/ml and 10 ng/ml) were added and cells were cultured for another 4 h. During the four-hour LPS simulation, the probiotics remained in the sample. Stimulation with LPS alone served as positive control.

### ROS production

For detection of ROS, CBMC were adjusted to 5 × 10^5^ cells/ml in RPMI 1640 and incubated with dihydrorhodamine 123 (DHR, Sigma) at a final concentration of 100 µM in PBS containing Ca^2+^ and Mg^2+^ for 15 minutes at 37 °C. After that, cells were washed, surface stained with anti-CD14 antibody (clone TÜK4, Miltenyi Biotech, Bergisch Gladbach, Germany) and ROS production was analysed by flow cytometry.

### Flow cytometry

Antibodies used for extracellular staining of monocytes and their surface molecules were purchased from BD Biosciences, Heidelberg, Germany (CD16-PE (clone 3G8), CD80-PE (clone L307.4), CD86-FITC (clone 2331), TLR-2-APC (clone 11G7)), TLR-4-PE (clone TF901)), Biolegend, San Diego (PD-L1-APC (clone 29E.2A3) and Miltenyi Biotech, Bergisch-Gladbach, Germany (CD11b-APC (clone REA713), CD14-PerCP (clone TÜK4) and CD18-FITC (clone TS1/18)). Supplementary Fig. 1A shows gating strategy for monocytes.

For intracellular staining, 1 × 10^6^ extracellular stained CBMC were washed with FACS-buffer (PBS with 0.1% foetal calf serum (FCS, Sigma, Munich, Germany) and 0.1% Na-azide (Sigma)). In all, 200 µl of Cytofix/Cytoperm (BD Biosciences) were added and cells incubated for 20 min at 4 °C. After that, cells were washed with Perm/Wash buffer (BD Biosciences) and intracellular antibodies (IL-1β-PE (clone AS10), IL-8-PE (clone G265-8), TNF-α-PE (clone 6401.111), TGF-β-PE (clone TW4-9E7), all from BD Biosciences) were added and incubated for 30 min at 4 °C. Cells were then washed twice with FACS-buffer and analysed.

Data acquisition was performed with a FACSCanto flow cytometer (BD Bioscience) and data analysed via FlowJo V10 (FlowJo, LLC, Ashland, OR).

### Statistics

Statistical analysis was done with GraphPad Prism version 9.1.2. Values were tested for Gaussian distribution using D’Agostino and Pearson omnibus normality test. Differences in MFIs (CD11b, CD18, CD86, TLR2, cytokine MFIs, ROS) were analysed using the paired t­test and differences in percentages (CD16, TLR-4 and PD-L1, cytokine percentages) were analysed using the Wilcoxon matched-paired signed-rank test. A *p* value < 0.05 was considered significant. *N*—numbers given in the figure captions are the numbers of independently performed experiments.

## Results

### Stimulation of cord blood monocytes with *Lactobacillus rhamnosus* induces a pro­inflammatory phenotype

**Fig. 1:**
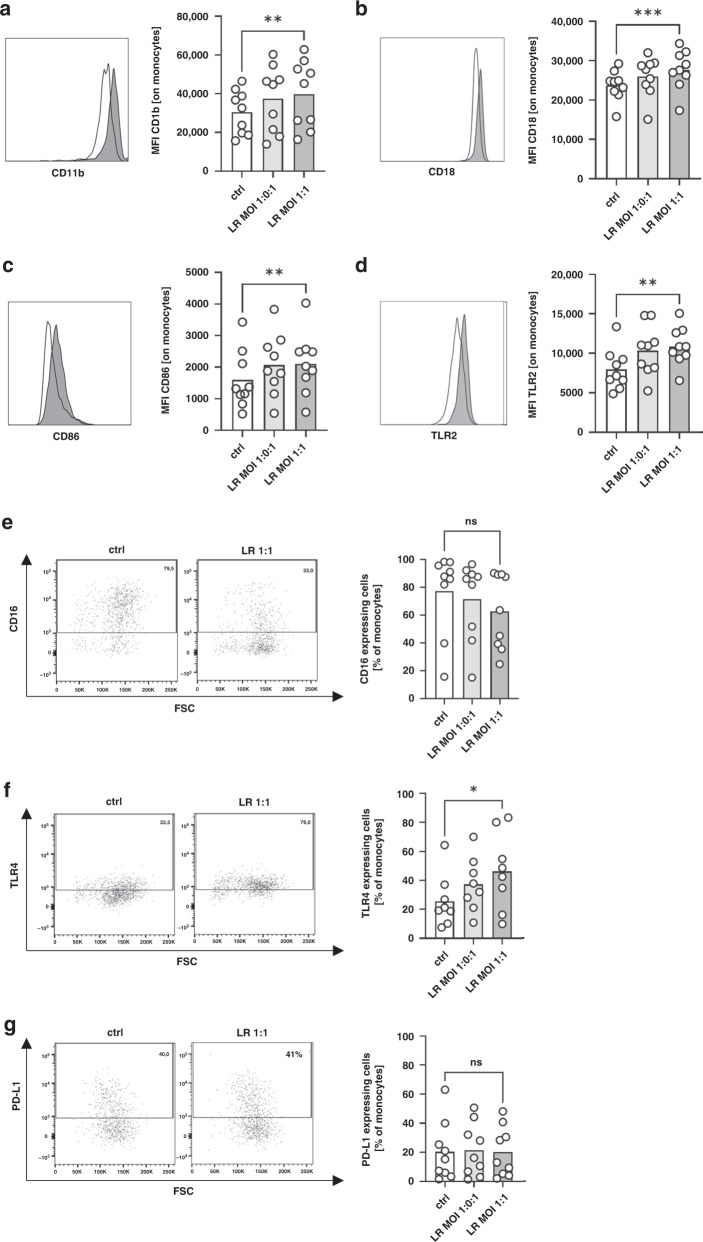
Expression of surface molecules on cord blood monocytes after stimulation with *Lactobacillus rhamnosus*. CBMC were isolated, cultured overnight and stimulated with *Lactobacillus rhamnosus* (LR) in a MOI of 1:0.1 and 1:1 for one hour. Expression of surface molecules CD11b, CD16, CD18, CD86, TLR2, TLR4 and PD-L1 was determined by flow cytometry. **a**–**d** Representative histograms show expression of CD11b (**a**), CD18 (**b**), CD86 (**c**) and TLR2 (**d**) on unstimulated monocytes (white) and monocytes after stimulation with LR in a MOI of 1:1 (grey) and scatter plots with bars show the mean fluorescent intensity (MFI) for expression of CD11b, CD18, CD86 and TLR2 on cord blood monocytes without stimulation (white bars) and after stimulation of LR in different MOIs (grey bars). *n* = 9, **p < 0.01, ***p < 0.001; paired *t* test. **e**–**g** Density plots show expression of CD16 (**e**), TLR4 (**f**) and PD-L1 (**g**) on unstimulated monocytes (ctrl) and monocytes after stimulation with LR in a MOI of 1:1 (LR 1:1) and scatter plots with bars show percentage of cord blood monocytes expressing CD16 (**e**), TLR4 (**f**) and PD-L1 (**g**) without stimulation (white bars) and after stimulation of LR in different MOIs (grey bars). *n* = 8–9, *p < 0.05, ns not significant; Wilcoxon matched-pairs signed-rank test.

First, we stimulated cord blood cells overnight with LR in a MOI of 1:0.1 and 1:1 and analysed expression of surface molecules on monocytes. All cord blood monocytes expressed integrins CD11b and CD18, the co-stimulatory molecule CD86 and the toll-like receptor 2 (TLR2), while only a part of cord blood monocytes expressed the Fc-receptor CD16, TLR4 and the co-inhibitory molecule programmed death ligand 1 (PD-L1). Stimulation with LR induced the expression of CD11b, CD18, CD86, TLR2 and TLR4 in a concentration-dependent on cord blood monocytes fashion (all *n* = 9, *p* < 0.05 for unstimulated cells versus MOI 1:1, Fig. 1a–d, f and Supplementary Table 1), while expression of CD16 and PD-L1 remained unchanged (Fig. 1e, g). After five hours of stimulation expression of CD11b and CD18 decreased in response to LR stimulation, while effects on the other surface markers examined remained similar to that after one hour (Supplementary Fig. 1B–H).

### Stimulation of cord blood monocytes with *Lactobacillus rhamnosus* induces the production of pro- and anti-inflammatory cytokines

**Fig. 2:**
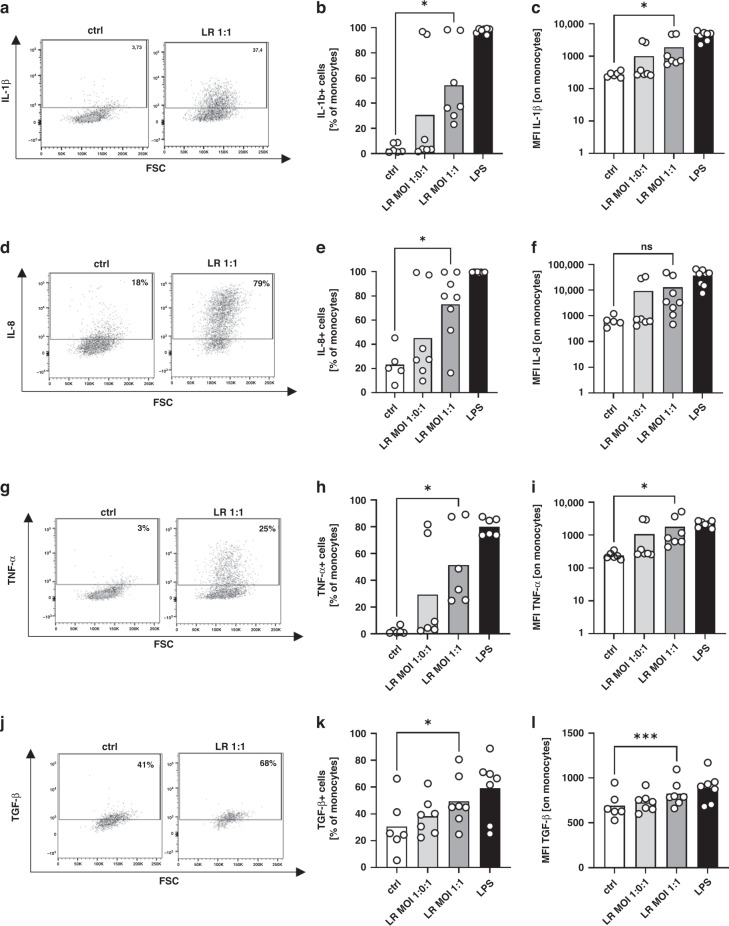
Expression of cytokines by cord blood monocytes after stimulation with *Lactobacillus rhamnosus*. CBMC were isolated, cultured overnight and stimulated with *Lactobacillus rhamnosus* (LR) in a MOI of 1:0.1 and 1:1 for one hour. Afterwards brefeldin was added and cells were cultured for another four hours. LPS stimulated cells served as control. Expression of IL-1β, IL-8, TNFα and TGF-β was determined after intracellular staining by flow cytometry. **a**, **d**, **g**, **j** Representative density plots show expression of IL-1β (**a**), IL-8 (**d**), TNF-α (**g**) and TGF-β (**j**) on unstimulated monocytes (ctrl) and monocytes after stimulation with LR in a MOI of 1:1 (LR 1:1). **b**, **e**, **h**, **k** Scatter plots with bars show percentages of cord blood monocytes expressing IL-1β (**b**), IL-8 (**e**), TNF-α (**h**) and TGF-β (**k**) without stimulation (white bars) and after stimulation of LR in different MOIs (grey bars) or after stimulation with LPS (black bars). **c**, **f**, **i**, **l** Scatter plots with bars show the mean fluorescent intensity (MFI) for expression of IL­1β (**c**), IL-8 (**f**), TNF-α (**i**) and TGF-β (**l**) in cord blood monocytes without stimulation (white bars) and after stimulation of LR in different MOIs (grey bars) or after stimulation with LPS (black bars). *n* = 5–7, *p < 0.05, ***p < 0.001, ns not significant; Wilcoxon matched-pairs signed-rank test (**b**, **e**, **h**, **k**) and paired *t* test (**c**, **f**, **i**, **l**).

Next, we asked whether cytokine expression of monocytes changes after stimulation with LR. We stimulated CBMC for one hour with LR in a MOI of 1:0.1 and 1:1 followed by a 4-h incubation with brefeldin to stop cytokine secretion. Stimulation with LPS was used as positive control. As observed for surface molecules, LR also induced expression of IL-1β, IL-8, TNF-α and TGF-β (*n* = 5–8, *p* < 0.05, Fig. 2 and Supplementary Table 2) in cord blood monocytes in a concentration-dependent manner. The anti-inflammatory IL-10 was nearly not expressed in monocytes in our study, neither unstimulated nor after LPS stimulation. Starting from this very low level, LR led to a slight induction in IL-10 expression in monocytes (Supplementary Fig. 2).

### Stimulation of cord blood monocytes with *Lactobacillus rhamnosus* induces ROS production

The production of ROS by monocytes is an important mechanism in their response to bacteria. Thus, we analysed whether stimulation with LR induces ROS production in cord blood monocytes. All monocytes, whether stimulated or not, produced ROS. Stimulation with LR led to a slight, but significant increase in ROS production of cord blood monocytes (MFI 996 ± 296 versus 1011 ± 280 (MOI 1:0.1) and 1267 ± 322 (MOI 1:1), *n* = 8, *p* < 0.01 for unstimulated cells versus MOI 1:1, Fig. 3).

**Fig. 3:**
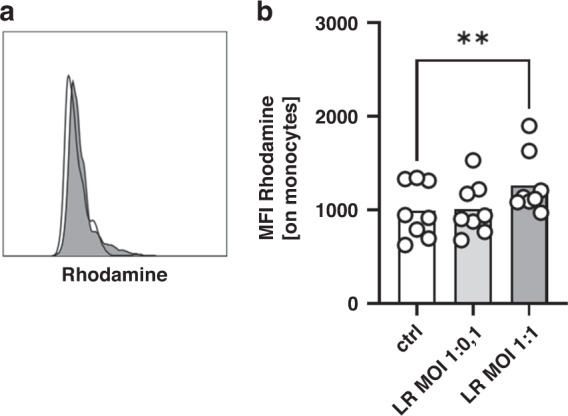
ROS production of cord blood monocytes after stimulation with *Lactobacillus rhamnosus*. CBMC were isolated, cultured overnight and stimulated with *Lactobacillus rhamnosus* (LR) in a MOI of 1:0.1 and 1:1 for 1 h. Production of ROS was assessed after staining with dihydrorhodamine (DHR) by flow cytometry. **a** Representative histogram shows expression of rhodamine in unstimulated monocytes (white) and monocytes after stimulation with LR (grey) in a MOI of 1:1. **b** Scatter plot with bars show the mean fluorescent intensity (MFI) for expression of Rhodamine in cord blood monocytes without stimulation (white bar) and after stimulation of LR in different MOIs (grey bars). *n* = 8, **p < 0.01, paired *t* test.

### Simultaneous stimulation of cord blood monocytes with *Lactobacillus rhamnosus* and LPS has additional effects on production of pro-inflammatory cytokines

**Fig. 4:**
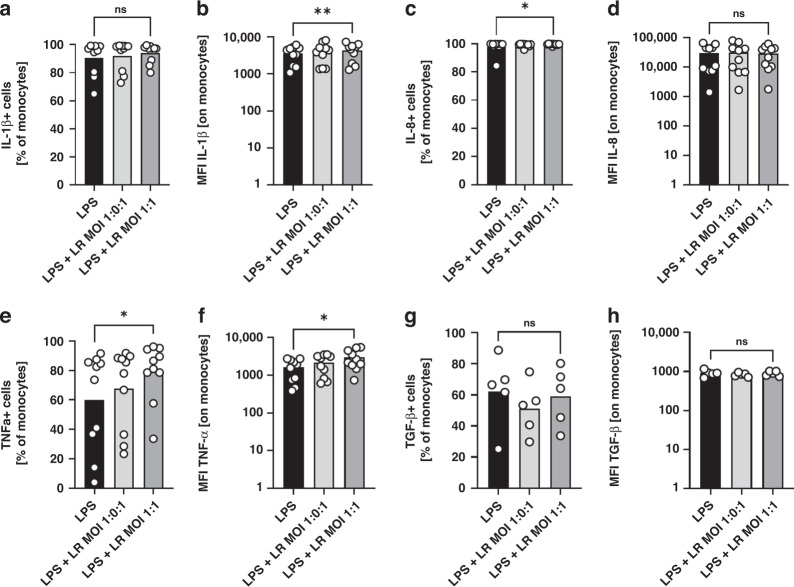
Expression of cytokines by cord blood monocytes after stimulation with *Lactobacillus rhamnosus* and LPS. CBMC were isolated, cultured overnight and stimulated with *Lactobacillus rhamnosus* (LR) in a MOI of 1:0.1 and 1:1 for one hour. Afterwards brefeldin and LPS were added and cells were cultured for another four hours. Cells stimulated with LPS alone served as control. Expression of IL-1β, IL-8, TNF-α and TGF-β was determined after intracellular staining by flow cytometry. **a**, **c**, **e**, **g** Scatter plots with bars show percentages of cord blood monocytes expressing IL­1β (**a**), IL-8 (**c**), TNF-α (**e**) and TGF-β (**g**) after stimulation with LPS alone (black bars) and after stimulation of LR in different MOIs (grey bars). **b**, **d**, **f**, **h** Scatter plots with bars show the mean fluorescent intensity (MFI) for expression of IL-1β (**b**), IL-8 (**d**), TNF-α (**f**) and TGF-β (**h**) in cord blood monocytes after stimulation with LPS alone (black bars) and after stimulation of LR in different MOIs (grey bars). *n* = 10, *p < 0.05, **p < 0.01, ns not significant; Wilcoxon matched-pairs signed-rank test (**a**, **c**, **e**, **g**) and paired *t* test (**b**, **d**, **f**, **h**).

Next, we asked whether LR might have an anti-inflammatory effect on monocytes when stimulated simultaneously with LPS. We again stimulated CBMC with LR for 1 h in a MOI of 1:0.1 and 1:1 but now added 1 µg/ml LPS followed by incubation with brefeldin to stop cytokine secretion. Since the most significant effects were observed for cytokine expression, we used this as the readout. Simultaneous stimulation of CBMC with LPS and LR also induced cytokine expression of IL-1β and TNF-α (*n* = 10, *p* < 0.05, Fig. 4a, b, e, f and Supplementary Table 3) but not of TGF-β (Fig. 4g, h and Supplementary Table 3) in cord blood monocytes compared to stimulation with LPS alone. Expression of IL-8 was already extremely high after LPS stimulation alone and stimulation with LR had no additional effect (Fig. 4c, d). Again, expression of IL-10 was very low, but simultaneous stimulation with LA and LPS induced IL-10 expression marginally (Supplementary Fig. 3A, B). A stimulatory effect of LR on cytokine production was also shown for lower LPS concentrations (100 and 10 ng/ml) (measured only for IL-8 and TNF-α, Supplementary Fig. 3C–F).

### Effects of stimulation with *Lactobacillus acidophilus* and *Bifidobacterium bifidum* on cytokine production of cord blood monocytes

**Fig. 5:**
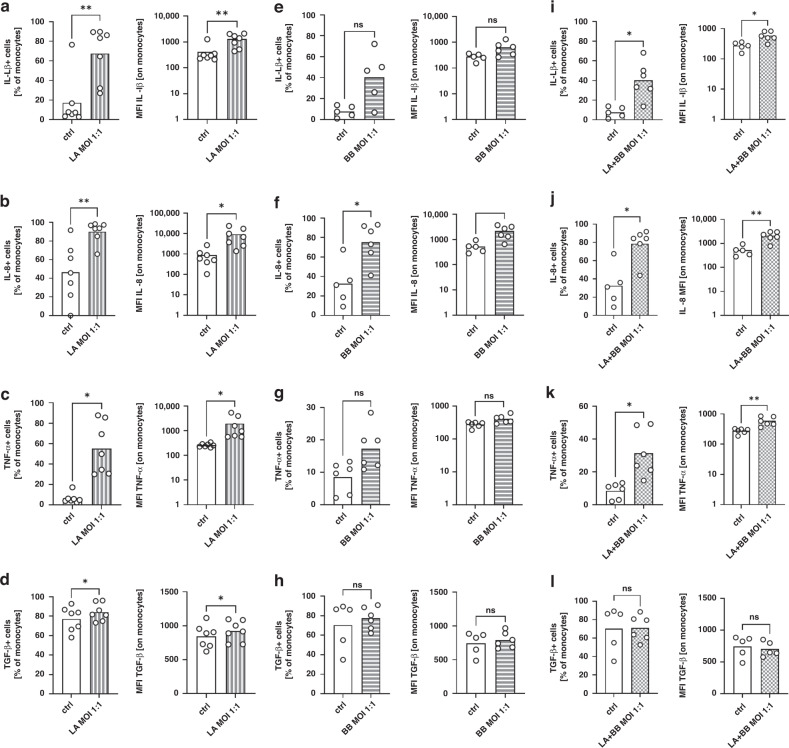
Expression of cytokines by cord blood monocytes after stimulation with *Lactobacillus acidophilus, Bifidobacterium bifidum* or *Lactobacillus acidophilus* and *Bifidobacterium bifidum* in combination. CBMC were isolated, cultured overnight and stimulated with *Lactobacillus acidophilus* (LA)*, Bifidobacterium bifidum* (BB) or *Lactobacillus acidophilus* and *Bifidobacterium bifidum* in combination (LA + BB) in a MOI of 1:0.1 and 1:1 for 1 h. Afterwards brefeldin was added and cells were cultured for another four hours. Cells stimulated with LPS alone served as control. Expression of IL-1β, IL-8, TNF-α and TGF-β was determined after intracellular staining by flow cytometry. **a**–**l** Scatter plots with bars show percentages of cord blood monocytes expressing and mean fluorescent intensity (MFI) for expression of IL-1β (**a**, **e**, **i**), IL-8 (**b**, **f**, **j**), TNF-α (**c**, **g**, **k**) and TGF-β (**d**, **h**, **l**) of unstimulated monocytes (white bars) and after stimulation with LA (**a**–**d**), BB (**e**–**h**) and LA + BB (**i**–**l**) in a MOI of 1:1 (striped/checked bars). *n* = 5­–7, *p < 0.05, **p < 0.01, ns not significant; Wilcoxon matched-pairs signed-rank test (for percentages) and paired *t* test (for MFIs).

Lastly, we analysed the effect of stimulation with two other probiotic strains, LA and BB alone or in combination, which are frequently used for the prevention of NEC in preterm infants, on cytokine expression of neonatal monocytes. As observed for LR, LA induced expression of all cytokines analysed in cord blood monocytes (*n* = 7, *p* < 0.05, Fig. 5a–d and Supplementary Table 4). In contrast, BB showed only a stimulatory effect on IL-8 expression (Fig. 5f and Supplementary Table 4), but not on the expression of IL-1β, TNF-α or TGF-β (Fig. 5e, g, h and Supplementary Table 4). Stimulation with a combination of LA and BB again induced the expression of IL-1β, IL-8 and TNF-α (*n* = 6, *p* < 0.05, Fig. 5i–k and Supplementary Table 4) but not the expression of TGF-β (Fig. 5l and Supplementary Table 4). Comparison of the relative induction of cytokine production by LA, BB, and the combination of both showed that after simultaneous stimulation, the levels of cytokine expression were similar to those of stimulation with BB alone, i.e., were lower than those of stimulation with LA alone (Supplementary Fig. 4). Simultaneous stimulation of CBMC with LPS and LA, BB or LA + BB had the same effect as simultaneous stimulation of CBMC with LPS and LR had, and induced the expression of IL-1β and TNF-α but not the expression of TGF-β and IL-8 (Supplementary Fig. 5).

## Discussion

Probiotics have been shown to have beneficial effects on a variety of diseases, especially inflammatory bowel disease and allergies.^14,15^ In preterm neonates, probiotics reduce the incidence of NEC and LOS.^18–20^ However, the mechanisms mediating these beneficial effects are incompletely understood. We here investigated the direct impact of different probiotic strains on neonatal monocyte phenotype and function in vitro and show that stimulation with LR, LA and BB induces pro-inflammatory responses in neonatal monocytes, both alone and in combination with LPS.

Our first finding of increased expression of activation markers on neonatal monocytes after stimulation with LR is in line with previous results reporting an induction of activation markers on human dendritic cells (DCs) by probiotic strains.^32–34^ Corresponding to the increased expression of CD11b and CD18 observed in our experiments, it was shown that stimulation with *Lactobacilli* induced phagocytosis of macrophages in vitro^35,36^ and that feeding mice with *Lactobacilli* induced phagocytic capacity in peritoneal and alveolar macrophages^37,38^ suggesting an improvement in infection resistance by probiotics. The decreased expression of CD11b and CD18—receptors directly involved in phagocytosis—after longer stimulation with LR may be due to internalisation during LR phagocytosis. The increased expression of TLR2 observed here is in line with a recent study analysing gene expression in human monocytes after stimulation with *Lactobacilli*^39^; however, those authors only detected an upregulation of TLR2, but not TLR4 gene expression upon probiotic stimulation.

Further, we found an increased expression of IL-1β, IL-8, TNF-α and TGF-β and to a small extend IL-10 after stimulation with LR. A number of previous studies have already investigated cytokine expression of adult monocytes or mononuclear cells after stimulation with different probiotic strains. All, including ours, showed an up-regulation of pro- and anti-inflammatory cytokines by probiotics.^40–43^ Only one study examined neonatal cells and also showed an increase in cytokine production, albeit to a lesser extent than found for *E. coli.*^44^

The effects on cytokine production after stimulation with LR were also present following additional stimulation with LPS. These in vitro effects are in contrast with those of Hart et al. showing decreased LPS-induced pro-inflammatory cytokine expression in lamina propria intestinal mononuclear cells following incubation with probiotics. It could be speculated that in lamina propria cells, unlike cord blood cells, priming with bacterial antigens has already taken place, leading to the observed differences. Similarly, several in vivo studies showed an anti-inflammatory effect. In asthmatic mice, for example, oral treatment with probiotics reduced inflammatory cytokine expression in bronchoalveolar lavage fluid.^45^ Similar observations were made in septic mice, where treatment with LR decreased mortality and expression of pro-inflammatory cytokines^46^ and in mice with experimentally induced colitis, where extracellular vesicles from *Lactobacillus plantarum* reduced inflammatory cytokine expression in colonic tissue.^47^ Interestingly, Flinterman et al. observed opposing effects of probiotics after in vitro stimulation and after oral administration. After in vitro stimulation with a probiotic mixture, they found, corresponding to our data, increased production of several cytokines, especially monocyte cytokines. However, after oral administration for three months, ex vivo stimulated immune cells showed decreased cytokine production. They speculated that an explanation for this might be the promotion of gut barrier functions by probiotics when administered in vivo leading to a diminished bacterial translocation and decreased activation of monocytes in the circulation.^48^ In addition, it is likely that in vivo complex interactions with other cell types—not only immune cells—lead to changes in the transcriptional profile of monocytes, which in turn may cause them to respond differently to bacterial stimulation.

We observed an increase in ROS production by neonatal monocytes upon stimulation with LR. This is in line with a recent study showing an increase in ROS production by macrophages from children stimulated with *Lactobacillus casei.*^35^ In addition, the study showed improved intracellular killing of bacteria after probiotic stimulation, suggesting that probiotics improve infection resistance. Contrary to our results, several Lactobacilli strains have been shown to have antioxidant capacity and to produce, for example, catalase, a ROS inhibitor.^49,50^ However, LR was not among the strains analysed.

Finally, we investigated two other probiotic strains alone and in combination for their effect on cytokine expression and found that both LA and BB induced production of IL-1β, IL-8, TNF­α and TGF-β either when used alone or in combination with LPS, albeit to a lesser extend for BB. In combination, LA and BB stimulated cytokine production similar to BB alone suggesting that BB attenuates the pro-inflammatory effects of LA. It has been known for some time that the beneficial properties of probiotics are strain-specific. For example, probiotics that induce IL-10 in vitro have been shown to have the most beneficial effects on acute colitis in vivo.^51^ Even if an in vitro model only inadequately reflects the situation in vivo, where probiotics interact not only with individual immune cells but also with other cell types, in vitro models with probiotics and immune cells seem to be suitable as a screening method for potentially beneficial effects.^52,53^ Previous studies with adult immune cells showed that *Lactobacilli* induce both pro- and anti-inflammatory cytokines, while *Bifidobacterium* strains preferentially induce anti-inflammatory IL-10.^54,55^ This is in line with our results where BB showed significantly less stimulatory effects on proinflammatory cytokines than LR and LL; however, due to the very low expression of IL-10 in our experiments with LR, we did not analyse IL-10 expression after BB and LA stimulation. Further studies are needed to investigate whether the strain-specific differences observed in adult immune cells apply accordingly in neonatal immune cells.

It has to be mentioned here that we used very low concentrations of probiotics (MOI 1:0.1 and MOI 1:1) for our experiments. At higher concentrations, there was first a pronounced shedding of CD14, so that the identification of monocytes was no longer possible, and then the cells died. Evrard et al. showed strong dose-dependent effects of LR on dendritic cells using a range of bacterial concentrations from MOI 0.01 to MOI 100.^32^ We suspect that the toxic effects we observed are due to different bacterial processing and culture conditions.

A limitation of our study is that we only investigated the impact of probiotics on cord-blood monocytes but not monocytes from peripheral blood of preterm or newborn infants. Due to the normally non-existent exposure to microorganisms in utero in contrast to the postnatal situation where contact with billions of microorganisms occurs very rapidly, it is possible that monocytes from cord blood respond differently to stimulation with probiotics than monocytes from peripheral blood. Due to the very small amounts of blood available in newborns and especially in premature infants, it is very difficult to perform extensive studies such as those performed here on this material. In addition, probiotics are often administered immediately postnatally and it is therefore clinically relevant how previously unexposed monocytes react to them. Similar studies as the one shown here on postnatal blood samples of newborns and premature infants would be desirable to obtain a more complete picture of the effect of probiotics. Furthermore, the cord blood donation in our study was anonymous, so that we could not collect any data, e.g. on maternal allergies, which could possibly have an influence on the results.^56^ We selected specific target parameters, such as expression of activation-associated surface markers, production of pro- and anti-inflammatory cytokines, and ROS production. However, these parameters by far do not cover all functions of monocytes. Again, further studies would be desirable, for example, to investigate the effect of probiotics on other monocyte functions such as phagocytosis or antigen presentation.

In summary, we show here that probiotics (in our case LR, LA and BB) induce pro­inflammatory effects in neonatal immune cells similar to those seen in adult immune cells, thereby possibly improving the ability to fight infection. Further studies are necessary to investigate the effects of different probiotic strains on neonatal immune cells in a more differentiated manner, in order to obtain indications as to which strains are best suited for the prevention of inflammatory diseases in the neonatal period such as sepsis, NEC or BPD.

## Supplementary information

Supplementary Figures_Mono and probiotics in vitro_Revision

Supplementary Tables_monos-probiotics

## Data Availability

The datasets generated during and/or analysed during the current study are available from the corresponding author on reasonable requests.
