# Supplementary material for: Different probiotic strains alter human cord blood monocyte responses
Source: Pediatr Res. 2022 Dec 7;94(1):103–11. doi: 10.1038/s41390-022-02400-5 (PMC10356588; doi:10.1038/s41390-022-02400-5)
Supplement: Supplementary file 1 — Supplementary Figures_Mono and probiotics in vitro_Revision [file 41390_2022_2400_MOESM1_ESM.pdf]

Supplementary Figure 1

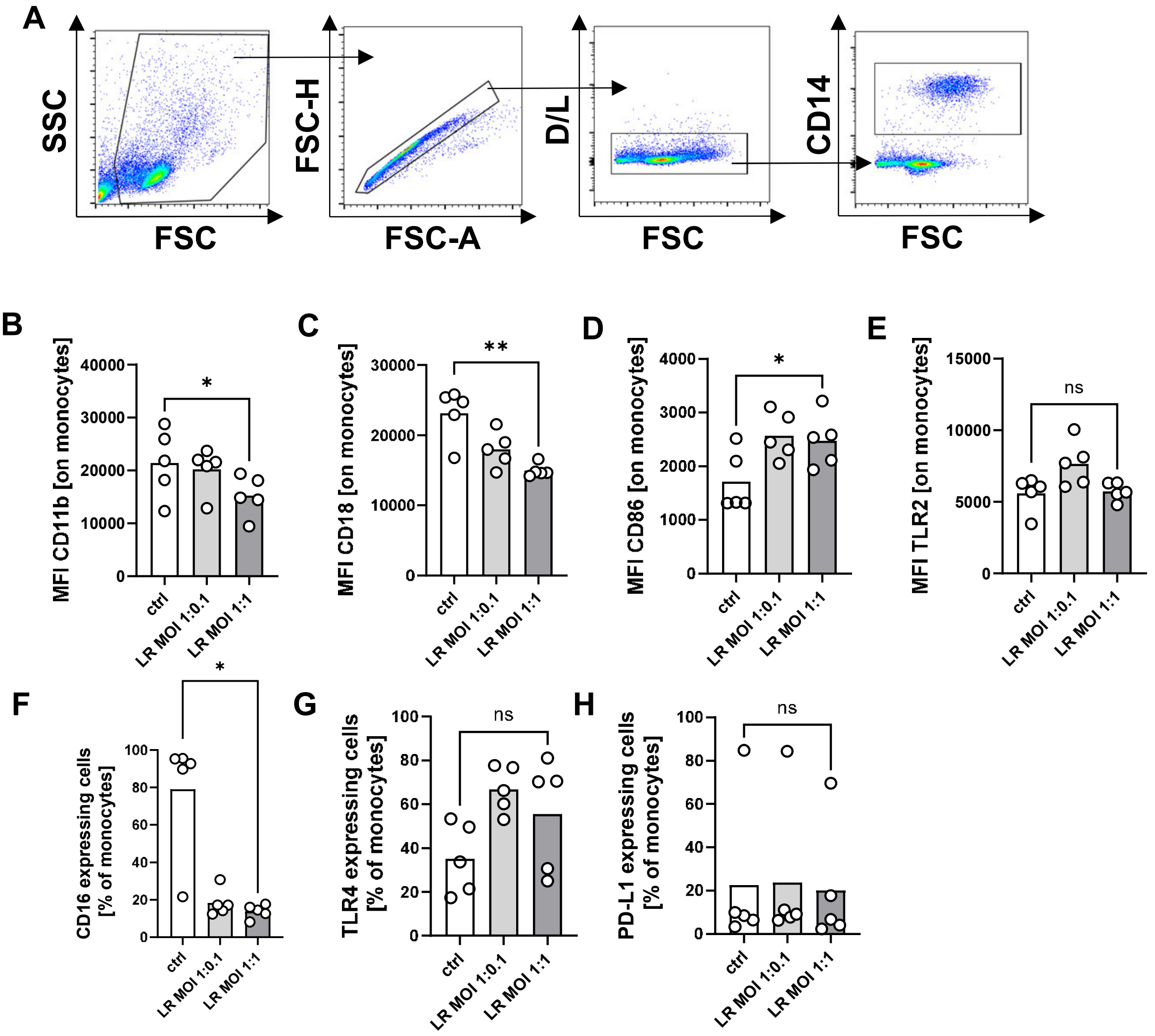

Supplementary Figure 1: Expression of surface molecules on cord blood monocytes after stimulation with *Lactobacillus rhamnosus*

CBMC were isolated, cultured overnight and stimulated with *Lactobacillus rhamnosus* (LR) in a MOI of 1:0.1 and 1:1 for five hours. Expression of surface molecules CD11b, CD16, CD18, CD86, TLR2, TLR4 and PD-L1 was determined by flow cytometry. (A) Representative density plots show gating strategy of monocytes (B-H) Scatter plots with bars show the mean fluorescent intensity (MFI) for expression of CD11b, CD18, CD86 and TLR2 on cord blood monocytes (B-E) and percentage of cord blood monocytes expressing CD16, TLR4 and PD-L1 (F-H) without stimulation (white bars) and after stimulation of LR in different MOIs (grey bars). * $p<0.05$, n=8-9, ** $p<0.01$, *** $p<0.001$; ns not significant; paired t-test for B-E and Wilcoxon matched pairs signed rank test for F-H.

Supplementary Figure 2

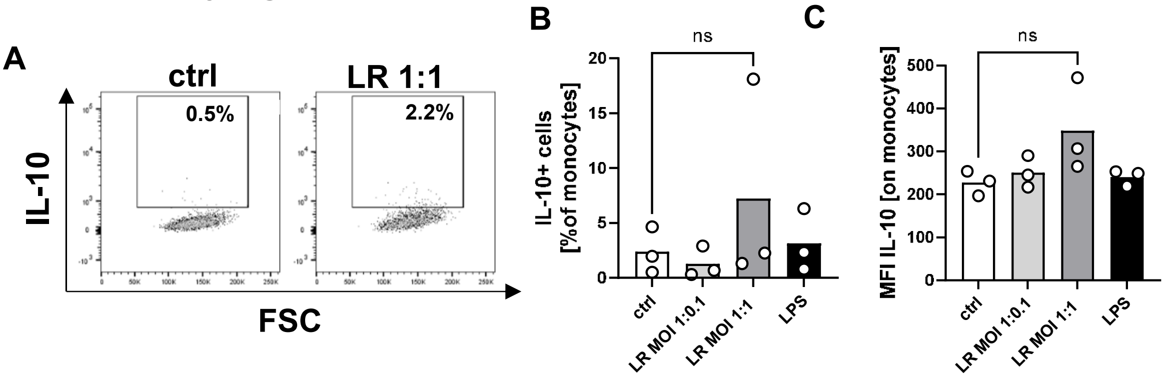

Supplementary Figure 2: Expression of IL-10 by cord blood monocytes after stimulation with *Lactobacillus rhamnosus*

CBMC were isolated, cultured overnight and stimulated with *Lactobacillus rhamnosus* (LR) in a MOI of 1:0.1 and 1:1 for one hour. Afterwards brefeldin was added and cells were cultured for another four hours. LPS stimulated cells served as control. Expression of IL-10 was determined after intracellular staining by flow cytometry. (A) Representative density plots show expression of IL-10 on unstimulated monocytes (ctrl) and monocytes after stimulation with LR in a MOI of 1:1(LR 1:1). (B+C) Scatter plots with bars show percentages of cord blood monocytes expressing IL-10 (B) mean fluorescent intensity (MFI) for expression of IL-10 (C) without stimulation (white bars) and after stimulation of LR in different MOIs (grey bars) or after stimulation with LPS (black bars). n=3, ns not significant; Wilcoxon matched pairs signed rank test (B) and paired t-test (C).

Supplementary Figure 3

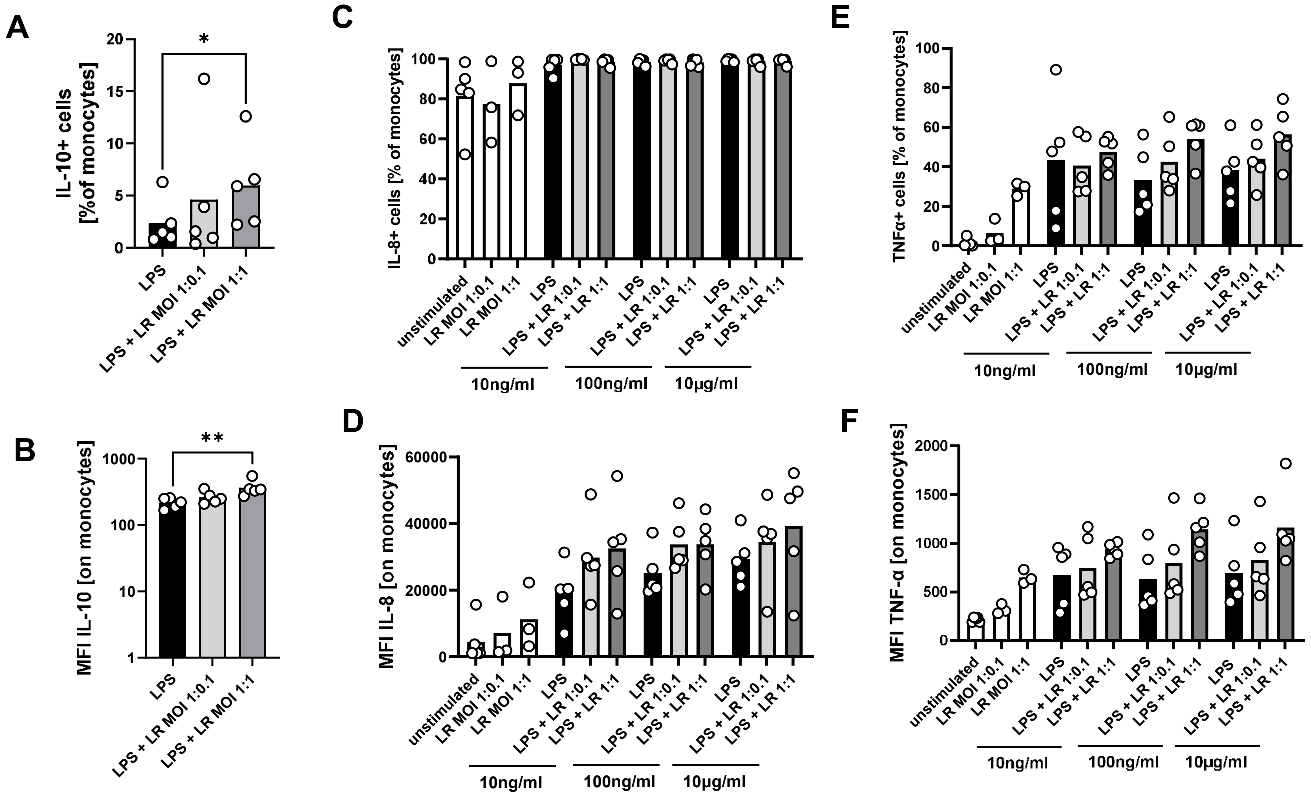

Supplementary Figure 3: Expression of cytokines by cord blood monocytes after stimulation with *Lactobacillus rhamnosus* and LPS

CBMC were isolated, cultured overnight and stimulated with *Lactobacillus rhamnosus* (LR) in a MOI of 1:0.1 and 1:1 for one hour. Afterwards brefeldin and LPS in different concentrations were added and cells were cultured for another four hours. Cells stimulated with LPS alone served as control. Expression of IL-8, IL-10 and TNF-α was determined after intracellular staining by flow cytometry. (A+B) Scatter plots with bars show percentages of cord blood monocytes expressing IL-10 (A) and mean fluorescent intensity (MFI) for expression of IL-10 (B) after stimulation with LPS alone (black bars) and after stimulation of LR in different MOIs (grey bars). (C-F) Scatter plots with bars show percentages of cord blood monocytes expressing IL-8 (C) and TNF-α (E) and mean fluorescent intensity (MFI) for expression of IL-8 (D) and TNF-α (F) after stimulation without LPS and LR (white bars) and after stimulation with LPS in different concentrations (black bars) and with or without LR (grey bars). n=3-5.

Supplementary Figure 4

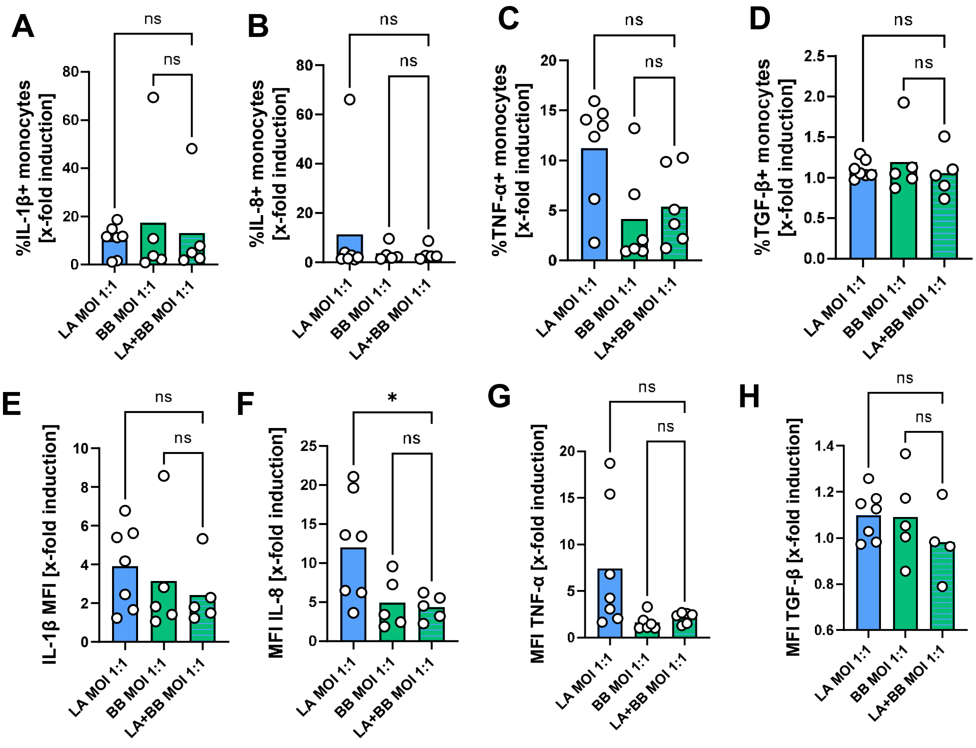

Supplementary Figure 4: Expression of cytokines by cord blood monocytes after stimulation with *Lactobacillus acidophilus, Bifidobacterium bifidum* or *Lactobacillus acidophilus* and *Bifidobacterium bifidum* in combination

CBMC were isolated, cultured overnight and stimulated with *Lactobacillus acidophilus* (LA)*, Bifidobacterium bifidum* (BB) or *Lactobacillus acidophilus* and *Bifidobacterium bifidum* in combination (LA+BB) in a MOI of 1:0.1 and 1:1 for one hour. Afterwards brefeldin was added and cells were cultured for another four hours. Expression of IL-1β, IL-8, TNF-α and TGF-β was determined after intracellular staining by flow cytometry. (A-L) Scatter plots with bars show relative induction of percentages of cord blood monocytes expressing and mean fluorescent intensity (MFI) for expression of IL-1β (A, E), IL-8 (B, F), TNF-α (C, G) and TGF-β (D, H) of monocytes and after stimulation with LA (blue bars), BB (green bars) and LA+BB (blue-green striped bars) in a MOI of 1:1. n=5-7, *$p<0.05$, ns not significant; Wilcoxon matched pairs signed rank test (for A-D) and paired t-test (for E-H).

Supplementary Figure 5

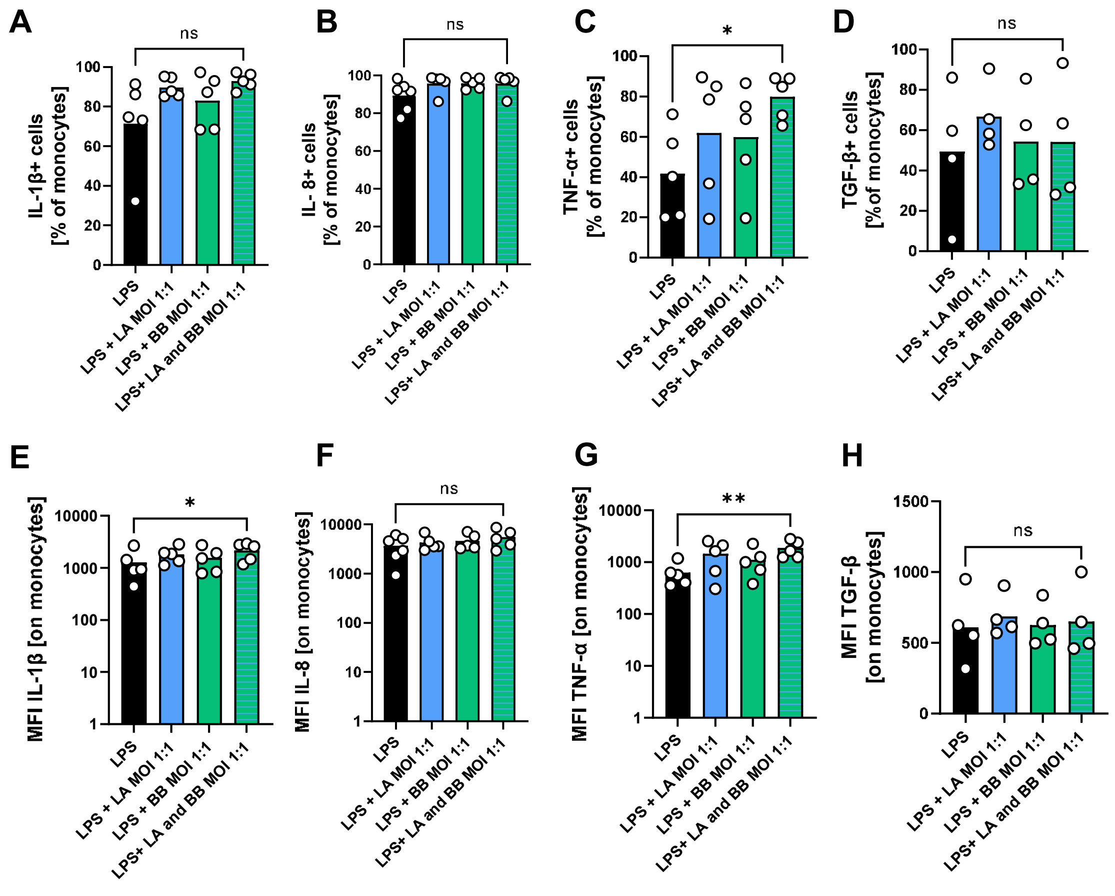

Supplementary Figure 5: Expression of cytokines by cord blood monocytes after simultaneous stimulation with LPS and *Lactobacillus acidophilus, Bifidobacterium bifidum* or *Lactobacillus acidophilus* and *Bifidobacterium bifidum* in combination

CBMC were isolated, cultured overnight and stimulated with *Lactobacillus acidophilus* (LA)*,* *Bifidobacterium bifidum* (BB) or *Lactobacillus acidophilus* and *Bifidobacterium bifidum* in combination (LA+BB) in a MOI of 1:0.1 and 1:1 for one hour. Afterwards 10µg/ml LPS and brefeldin was added and cells were cultured for another four hours. Expression of IL-1β, IL-8, TNF-α and TGF-β was determined after intracellular staining by flow cytometry. (A-L) Scatter plots with bars show relative induction of percentages of cord blood monocytes expressing and mean fluorescent intensity (MFI) for expression of IL-1β (A, E), IL-8 (B, F), TNF-α (C, G) and TGF-β (D, H) of monocytes and after stimulation with LPS alone (black bars), LPS and LA (blue bars), LPS and BB (green bars) and LPS and LA+BB (blue-green striped bars) in a MOI of 1:1. n=5-7, *p<0.05, **p<0.01, ns not significant; Wilcoxon matched pairs signed rank test (for A-D) and paired t-test (for E-H).
