# Supplementary material for: Different probiotic strains alter human cord blood monocyte responses
Source: Pediatr Res. 2022 Dec 7;94(1):103–11. doi: 10.1038/s41390-022-02400-5 (PMC10356588; doi:10.1038/s41390-022-02400-5)
Supplement: Supplementary file 2 — Supplementary Tables_monos-probiotics [file 41390_2022_2400_MOESM2_ESM.pdf]

Supplementary Table 1: Expression of surface molecules on cord blood monocytes after stimulation with *Lactobacillus rhamnosus*

	**CD11b (MFI)**	**CD16 (%)**	**CD18 (MFI)**	**CD86 (MFI)**	**TLR2 (MFI)**	**TLR4 (%)**	**PD-L1 (%)**
**ctrl**	30583 ± 11312	77.2 ± 29.2	23754 ± 3845	1598 ± 916	7964 ± 2586	25.7 ± 18.2	20.3 ± 20.2
**MOI 1:0.1**	37418 ±16963	71.4 ± 28.3	26005 ± 5046	2068 ± 978	10337 ± 3187	37.2 ± 18.7	21.5 ± 18.2
**MOI 1:1**	39801 ± 17421	62.8 ± 26.9	27709 ± 5030	2104 ± 976	10894 ± 2437	46.3 ± 26.7	20.2 ± 17.2
**p-value***	**<0.01**	0.05	**<0.001**	**<0.01**	**<0.01**	**<0.05**	0.65

* for ctrl vs. MOI 1:1

Supplementary Table 2: Cytokine expression in cord blood monocytes after stimulation with *Lactobacillus rhamnosus*

	**IL-1β**		**IL-8**		**TNF-α**		**TGF-β**	
	MFI	%	MFI	%	MFI	%	MFI	%
**ctrl**	276 ± 55	4.0 ± 3.6	673 ± 325	23.2 ± 14.4	243 ± 61	2.2 ± 2.4	691 ± 146	30.7 ± 20.9
**MOI 1:0.1**	1011 ± 1230	30.7 ± 44.5	9252 ± 14817	45.1 ± 37.3	1068 ± 1332	29.4 ± 38.2	728 ± 105	38.3 ± 13.8
**MOI 1:1**	1894 ± 1991	54.2 ± 31.6	12903 ± 18668	73.2 ± 27.9	1797 ± 1865	51.5 ± 29.9	824 ± 143	49.4 ± 18.9
**p-value***	**<0.05**	**<0.05**	0.06	**<0.05**	**<0.05**	**<0.05**	**<0.001**	**<0.05**

* for ctrl vs. MOI 1:1

Supplementary Table 3: Cytokine expression in cord blood monocytes after stimulation with *Lactobacillus rhamnosus* and LPS

	**IL-1β**		**IL-8**		**TNF-α**		**TGF-β**	
	MFI	%	MFI	%	MFI	%	MFI	%
**ctrl**	3680 ± 1768	90.6 ± 12.1	29598 ± 24679	97.8 ± 4.8	1610 ± 940	60.2 ± 33.1	913 ± 174	62.4 ± 23.2
**MOI 1:0.1**	4172 ± 2423	92.0 ± 10.5	31481 ± 27732	99.1 ± 1.3	2131 ± 1205	67.7 ± 27.5	831 ± 93	51.0 ± 17.0
**MOI 1:1**	4353 ± 2121	94.0 ± 6.8	28824 ± 20412	99.5 ± 0.6	2962 ± 1652	79.2 ± 19.7	899 ± 127	59.1 ± 19.1
**p-value***	**<0.01**	0.3	0.75	**<0.05**	**<0.05**	**<0.05**	0.78	0.50

* for ctrl vs. MOI 1:1

Supplementary Table 4: Cytokine expression in cord blood monocytes after stimulation with *Lactobacillus acidophilus* and *Bifidobacterium bifidum*

	**IL-1β**		**IL-8**		**TNF-α**		**TGF-β**	
	MFI	%	MFI	%	MFI	%	MFI	%
**LA ctrl**	417 ± 379	17.2 ± 26.6	903 ± 867	46.6 ± 30.9	260 ± 45	6.5 ± 4.9	913 ± 174	77.2 ± 12.6
**LA MOI 1:1**	1302 ± 684	67.5 ± 27.3	9302 ± 8279	90.1 ± 11.5	1943 ± 1928	55.4 ± 25.4	899 ± 127	84.1 ± 9.1
**p-value**	**<0.01**	**<0.01**	**<0.05**	**<0.01**	**<0.05**	**<0.01**	**<0.05**	**<0.05**
**BB ctrl**	274 ± 81	7.7 ± 5.3	526 ± 258	32.5 ± 22.3	278 ± 52	8.5 ± 4.8	743 ± 171	70.4 ± 24.3
**BB MOI 1:1**	637 ± 382	40.3 ± 25.0	2181 ± 1197	75.0 ± 20.0	414 ± 120	17.4 ± 6.5	790 ± 119	77.4 ± 11.5
**p-value**	0.05	0.06	**<0.05**	**<0.05**	0.05	0.08	0.22	0.31
**LA+BB ctrl**	274 ± 81	7.7 ± 5.3	526 ± 258	32.5 ± 22.3	278 ± 52	8.5 ± 4.8	743 ± 171	70.4 ± 24.3
**LA+BB MOI 1:1**	293 ± 117	40.4 ± 18.6	858 ± 868	41.4 ± 27.0	280 ± 93	9.6 ± 6.7	696 ± 176	63.0 ± 16.7
**p-value**	**<0.05**	**<0.05**	**<0.01**	**<0.05**	**<0.01**	**<0.05**	0.3	0.50
